# Voluntary Medical Male Circumcision: Strategies for Meeting the Human Resource Needs of Scale-Up in Southern and Eastern Africa

**DOI:** 10.1371/journal.pmed.1001129

**Published:** 2011-11-29

**Authors:** Kelly Curran, Emmanuel Njeuhmeli, Andrew Mirelman, Kim Dickson, Tigistu Adamu, Peter Cherutich, Hally Mahler, Bennett Fimbo, Thembisile Khumalo Mavuso, Jennifer Albertini, Laura Fitzgerald, Naomi Bock, Jason Reed, Delivette Castor, David Stanton

**Affiliations:** 1 Jhpiego, Baltimore, Maryland, United States of America; 2 International Health Department, Johns Hopkins Bloomberg School of Public Health, Baltimore, Maryland, United States of America; 3 United States Agency for International Development, Washington, District of Columbia, United States of America; 4 World Health Organization, Geneva, Switzerland; 5 National AIDS and STI Control Programme, Ministry of Health, Nairobi, Kenya; 6 Jhpiego/Tanzania, Dar es Salaam, Tanzania; 7 Ministry of Health, Dar es Salaam, Tanzania; 8 Ministry of Health, Mbabane, Swaziland; 9 Jhpiego/Swaziland, Mbabane, Swaziland; 10 Centers for Disease Control and Prevention, Atlanta, Georgia, United States of America; Centers for Disease Control and Prevention, United States of America

## Abstract

Voluntary medical male circumcision (VMMC) reduces female-to-male HIV transmission by approximately 60%; modeling suggests that scaling up VMMC to 80% of men 15- to 49-years-old within five years would avert over 3.3 million new HIV infections in 14 high priority countries/regions in southern and eastern Africa by 2025 and would require 20.33 million circumcisions. However, the shortage of health professionals in these countries must be addressed to reach these proposed coverage levels. To identify human resource approaches that are being used to improve VMMC volume and efficiency, we looked at previous literature and conducted a program review. We identified surgical efficiencies, non-surgical efficiencies, task shifting, task sharing, temporary redeployment of public sector staff during VMMC campaign periods, expansion of the health workforce through recruitment of unemployed, recently retired, newly graduating, or on-leave health care workers, and the use of volunteer medical staff from other countries as approaches that address human resource constraints. Case studies from Kenya, Tanzania, and Swaziland illustrate several innovative responses to human resource challenges. Although the shortage of skilled personnel remains a major challenge to the rapid scale-up of VMMC in the 14 African priority countries/regions, health programs throughout the region may be able to replicate or adapt these approaches to scale up VMMC for public health impact.

## Abstract 2

Kelly Curran and colleagues conducted a program review to identify human resource approaches that are being used to improve voluntary medical male circumcision volume and efficiency, identifying several innovative responses to human resource challenges.

**Summary Points**

- Scaling up voluntary medical male circumcision (VMMC) could avert millions of HIV infections in southern and eastern Africa, but shortages of health professionals are likely to limit progress.
- Potential responses to this human resource challenge include task shifting, task sharing, temporary redeployment of public sector staff during VMMC campaigns, expansion of the health workforce through recruitment of unemployed, recently retired, newly graduated, or on-leave health care workers, and the use of foreign volunteer medical staff.
- Approaches to solving the human resource challenge associated with VMMC scale-up that have already been implemented include the following: moving public sector clinicians to high-volume VMMC sites during campaigns (Tanzania), empowering nurses to conduct VMMC surgery (Kenya), and identifying untapped reserves of qualified nurses (Swaziland).
- These approaches provide models for other countries to replicate and adapt.
- Importantly, the effect of VMMC scale-up on other health services is not known and must be investigated in future studies.

## Introduction

Observational and experimental studies have shown that volunteer medical male circumcision (VMMC) reduces HIV acquisition among heterosexual men by approximately 60% [1]–[4]. In 2007, after the completion of three randomized controlled trials on VMMC, the World Health Organization (WHO) and the Joint United Nations Programme on HIV/AIDS (UNAIDS) recommended that VMMC should be part of comprehensive HIV prevention programming in regions with a generalized HIV epidemic and a relatively low level of male circumcision (MC). They also prioritized 13 countries in southern and eastern Africa (Table 1) for VMMC scale-up [2]–[5]. In addition to surgical intervention, WHO recommended that VMMC service packages should include HIV and MC education, HIV testing and counseling (HTC), screening for sexually transmitted infections, condom promotion and postoperative care. The United States President's Emergency Plan for AIDS Relief (PEPFAR) currently provides funding and technical support for implementing VMMC services in the 13 priority countries plus the Gambella National Regional State in Ethiopia.

**Table 1:** Density of physicians and nurses/midwives in VMMC priority countries.

Country	Physician Density (per 1,000 Individuals)	Nurse/Midwife Density (per 1,000 Individuals)	Year
Botswana	0.40	2.65	2004
Ethiopia^a^	0.02	0.24	2007
Kenya^a^	0.14	1.18	2002
Lesotho	0.05	0.62	2003
Malawi	0.02	0.28	2008
Mozambique	0.03	0.31	2006
Namibia	0.30	3.06	2004
Rwanda	0.02	0.45	2005
South Africa	0.77	4.08	2004
Swaziland	0.16	6.30	2004
Tanzania	0.01	0.24	2006
Uganda	0.12	1.31	2005
Zambia	0.06	0.71	2006
Zimbabwe	0.16	0.72	2004
Mean^b^	0.16	1.58	—
United States	2.56	9.37	2000

Sources: [32],[33].

a VMMC programming focuses on the Gambella Region of Ethiopia and the Nyanza Province of Kenya, but these data refer to health care densities over the whole country in both cases.

b Mean density of African-country-specific data is from different years.

**Figure 1:**
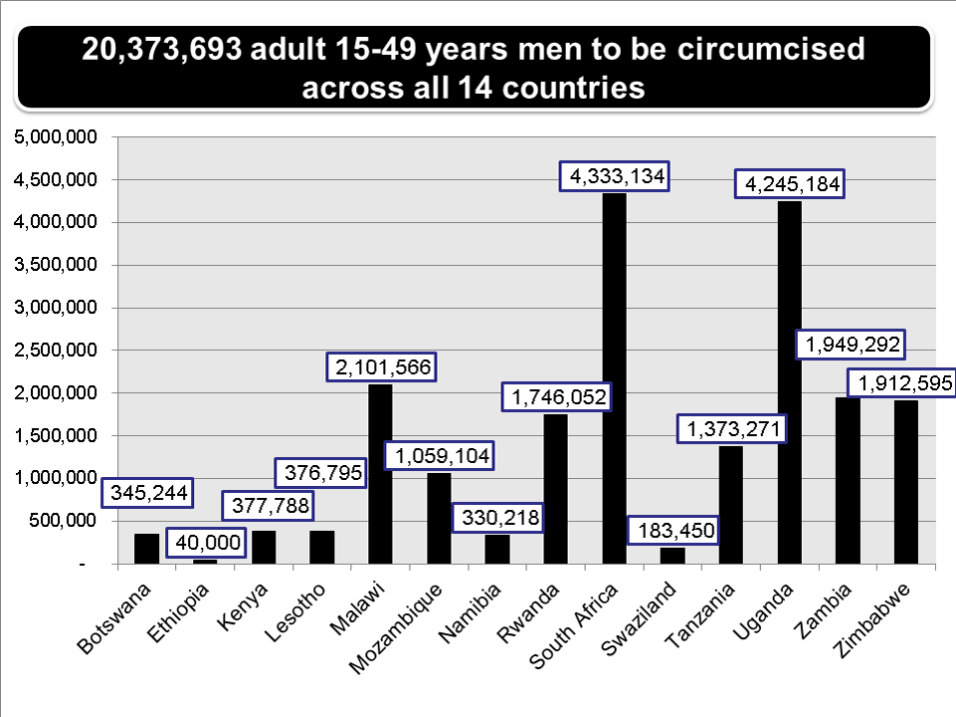
Number of circumcisions among men aged 15 to 49 years needed to reach 80% coverage in each of 14 priority countries/regions within five years. For Ethiopia (Gambella Region) and Kenya (Nyanza Province), only one region or province with low circumcision rates and high HIV prevalence is included, as most men in these countries are already circumcised.

Mathematical modeling studies suggest that to reach 80% VMMC coverage in the priority countries within five years would entail performing 20.33 million circumcisions between 2011 and 2015 (Figure 1); sustaining 80% coverage thereafter (universal coverage) would require an additional 8.42 million circumcisions between 2016 and 2025 [6]. Modeling also suggests that approximately 3.36 million new HIV infections and 386,000 AIDS deaths would be averted through 2025 at an estimated total cost of US$2,000,000,000; net savings would be US$16,550,000,000 [6].

Unfortunately, the number of clinicians, counselors, and support staff needed to achieve and sustain universal coverage of VMMC is expected to exceed the available human resources for health (HRH) in many African countries. WHO estimates that sub-Saharan Africa has 25% of the world's disease burden and 3% of the world's health workforce [7],[8]. As a consequence, sub-Saharan Africa needs to triple its health workforce in order to meet existing health needs [9]. Moreover, of the 57 countries globally with HRH crises, 36 are on the African continent [10].

More specifically, the southern and eastern African countries prioritized for VMMC scale-up for HIV prevention have low physician and nurse/midwife densities (Table 1) and very limited numbers of surgical specialists. For example, in 2008, there were only 75 surgeons and ten physician anesthetists serving Uganda's population of 27 million [11]. These countries also have heavy burdens of tuberculosis and other diseases that stretch their health systems [12] and that directly impact on their limited health workforce [13]. Finally, out-migration to higher-resource settings and economic limitations within the public sector further deplete HRH in these countries [14],[15].

Because of these HRH concerns, health ministries in the priority countries for VMMC scale-up have highlighted the possible disruption to other key HIV, primary care, and surgical services as major barriers to planning for or implementing the scale-up of VMMC services. In this review, we describe three approaches to optimizing HRH and minimizing disruptions to existing health services that have already been used in the implementation and scale-up of VMMC for public health impact.

### How Are HRH Limitations Being Addressed?

To determine the status of HRH in the southern and eastern African VMMC priority settings and to identify innovations in HRH that address resource challenges in VMMC, we examined policy and program reports from the 13 priority countries identified by WHO plus the Gambella National Regional State in Ethiopia to identify programs that have augmented their health workforce to increase efficiencies. (The Gambella Region was included because PEPFAR supports a VMMC program there, where MC prevalence is low and HIV prevalence is three times the national average.) Specifically, we looked for evidence within national and regional programs of the use of staff to improve volume and efficiency, of a demonstrated capacity to scale up services, and of the institution of measures for optimizing quality and safety. We also looked for evidence that these HRH approaches had been implemented through the public sector because this is a prerequisite for achieving universal coverage. VMMC programs based in Kenya, Tanzania, and Swaziland emerged from this analysis as programs that were using innovative HRH approaches, so we examined these programs more closely as case studies.

From the three selected VMMC program case studies, we identified seven main policy and programmatic approaches being applied in southern and eastern Africa to address the HRH needs of VMMC scale-up while minimizing disruptions to other key health services: surgical efficiencies, non-surgical efficiencies, and five types of human resource efficiencies (task shifting, task sharing, temporary redeployment of public sector health care workers during VMMC campaign periods, targeted recruitment of unemployed, recently retired, newly graduating, and on-leave health care workers, and recruitment of volunteer health care workers).

### Surgical Efficiencies

In our review of VMMC programs, we identified surgical technique, team composition, and use of prepackaged surgical kits as three surgical efficiencies that decrease provider downtime and increase the number of VMMCs that can be performed by a surgical team while maintaining high quality.

All three techniques for adult circumcision—dorsal slit, forceps-guided, and sleeve resection—have similar safety profiles and are included in the WHO/UNAIDS/Jhpiego surgical manual [16]. However, the forceps-guided procedure is, on average, two minutes, 45 seconds, faster than the dorsal slit procedure, and seven minutes, 40 seconds, faster than sleeve resection [17]. Although the forceps-guided procedure can be less esthetically pleasing initially, after complete wound healing all the methods produce similar esthetic results [16]. Given the number of VMMCs that need to be completed for public health impact, even relatively small time savings per procedure can have significant effects on the number of procedures completed [6],[18].

**Figure 2:**
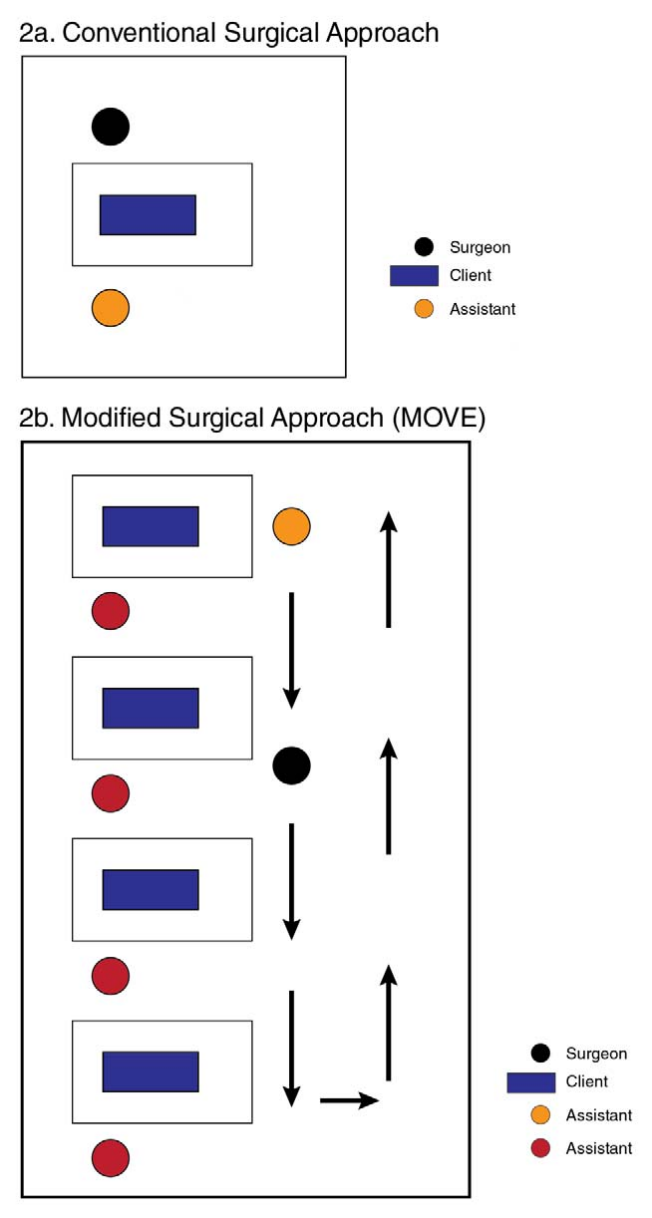
Conventional and modified surgical approaches to MC provision. (A) The conventional approach to MC provision requires 20–30 minutes per procedure and allows only two surgeries per hour (ten surgeries per day) because of the doctors “downtime” between procedures. (B) With the modified surgical approach to MC provision (MOVE), each procedure still takes 20–30 minutes, but task sharing reduces the doctor's time to 6–8 minutes per procedure, which allows eight surgeries per hour (40 surgeries per day).

Conventionally, adult and adolescent VMMC is provided by one doctor working with one assistant in an operating theater or procedure room. Using this model, a doctor–nurse surgical team can provide eight to ten VMMCs per day (Figure 2A). This conventional surgical model is inherently inefficient because with only one bed in use the doctor has “downtime” between clients. Time-and-motion studies conducted by the Bophelo Pele clinical team in Orange Farm, South Africa, showed that physician downtime can be almost eliminated by expanding the number of surgical bays (Figure 2B) and delegating many critical surgical tasks (including injection of local anesthesia and placement of simple interrupted sutures) to nurses (task sharing), thereby allowing doctors to focus on the highest-level skill steps (foreskin removal, hemostasis, and mattress sutures) [17]. This model is staff and resource intensive and is best suited for high-volume sites.

The Orange Farm team has also pioneered the use of prepackaged, fully disposable VMMC kits containing all of the instruments and consumables needed for one procedure. This innovation saves pre-procedure preparation time, and reduces time delays due to missing supplies or equipment. An assessment of minor surgical capacity in Mozambique, including the capacity to perform VMMC, showed that surgical supplies and equipment were often out of stock, and that this seriously undermined surgical productivity. Problems with water and electricity also impeded instrument processing in this setting [19]. The price of prepackaged surgical kits, which are particularly useful for high-volume sites, mobile outreach services, and other campaign-type settings, is a function of economies of scale. The pooled procurement of kits has already resulted in a reduction in market price from US$23 to US$11 per kit. Prepackaged kits also reduce the number of stock-outs and eliminate problems with instrument processing due to electricity outages. In program settings where reusable instruments continue to be used, pre-bundled packages can save time [20].

### Non-Surgical Efficiencies

A steady demand for VMMC services among males in the target age group is a necessary component for a successful program. Insufficient demand for supply is inefficient for the providers. Conversely, demand that exceeds services can create bottlenecks that impact program quality [21]. Supply and demand can be better matched by capturing information in real time on the number of clients, adverse events, and HIV testing uptake. Also, moving HTC and some other services (e.g., postoperative review) into the community during high-volume periods can improve client flow and optimize the use of the health workforce [21].

### Human Resource Efficiencies

Implementation of the five types of HRH efficiencies that we identified in our program review can improve both the volume and efficiency of VMMC services and can be adapted to the individual circumstances of a given health situation and setting.

Task shifting—the delegation of surgical steps to a trained non-physician clinician such as a nurse or clinical officer—can greatly expand the size of the workforce available to provide surgical services. Specifically, VMMC counseling and HTC can be delegated to lay counselors, and clinical officers and nurses, who are in greater supply in the workforce (Table 1), can replace doctors in all the steps in VMMC surgery. In task sharing, although some steps/procedures are delegated to a non-physician clinician such as a nurse, the highest-level skill steps (e.g., hemostasis) remain the physician's responsibility. Task sharing works well when combined with approaches to improve the layout and client flow of surgical services (e.g., the use of multiple beds). The expansion of the role of nurses in VMMC programs through both task shifting and task sharing has greatly improved program efficiency [22] and has important implications for countries at various stages of VMMC scale-up.

Another HRH efficiency is temporary redeployment of public sector staff involved in other aspects of health care to VMMC services during campaigns—redeployment is an approach that is commonly used during immunization campaigns. The use of a campaign model to provide high-volume VMMC services during periods of high demand (cold season, agriculturally slow periods, and school holidays) proved an effective strategy in Tanzania [21].

Targeted recruitment—the training and engagement of health care workers who are not currently active in the workforce such as part-time and unemployed workers, and recently retired or newly graduated nurses—is a good option for VMMC programs seeking long-term personnel. Objective quantification of existing HRH resources can identify some of these untapped HRH capacities, even in countries with documented HRH crises (see the Swaziland case study for more details). In addition, targeted short-term recruitment of on-leave nurses can be useful during campaigns.

Finally, the recruitment of volunteer health care workers from other countries during focused VMMC campaigns can temporarily increase the size of the health workforce. To date, Swaziland is the only country in the region that has recruited and deployed volunteer health care providers (specifically doctors) for VMMC services. This approach is a feasible and acceptable option in a country where the nursing scope of practice does not allow task shifting.

### Tanzania Case Study: Non-Surgical Efficiencies Optimize the Use of a Public Sector Health Workforce Deployed during Campaigns

 The Iringa Region has the highest adult HIV prevalence in Tanzania (15.7% compared to 5.7% nationwide) [23] and one of the lowest MC prevalence rates in the country (29%) [23]. The Tanzanian Ministry of Health, with PEPFAR support, began implementing VMMC in the Iringa Region in June 2010 in a six-week campaign during which 10,352 adult and adolescent men were circumcised across five sites [21]. Some HTC was done in the community, most providers came from the public sector, and task shifting allowed nurses and clinical officers to perform VMMC surgeries. Several additional non-surgical efficiencies were also implemented to match supply with demand for VMMC services.

On the demand side, June and July were selected for the campaign because this is a slow period on tea plantations, coincides with school holidays, and is the cool season, which the local population believes is associated with faster wound healing [24]. The program “harnessed” this period of naturally higher demand by the timely expansion of VMMC services while ensuring adequate client demand through multiple reinforcing messages to the population (e.g., radio advertisements, posters, loudspeaker announcements). Once it became clear that demand was extremely high, radio advertisements were halted.

On the supply side, to prevent a client-preparation bottleneck, HIV counselors from Ministry of Health sites and from PEPFAR-funded HTC programs were given one week's training in VMMC education and counseling before the campaign and then deployed during the campaign to provide VMMC counseling and HTC in tents pitched at the five VMMC sites and in the community. Community-based counselors referred interested clients to the nearest VMMC clinic using a referral card system. Community-based preoperative preparation of clients was also introduced. In addition, rather than being given a specific appointment time, clients were typically given a date for their surgery. This advance scheduling approach helped to ensure that clinics received an adequate—but not overwhelming—number of clients each day.

Another approach taken to match supply and demand was effective, motivational management of human resources. During the campaign, VMMC providers, counselors, and support staff typically worked ten-hour days, Monday through Friday, and six-hour days on Saturdays. To motivate personnel, the program provided T-shirts and daily meals. Moreover, site visits were conducted during the campaign to remind frontline providers that modeling studies suggest that, in Iringa, one HIV infection will be averted by 2025 for every 4.5 VMMCs done in the next five years. These reminders kept providers motivated and focused on the HIV prevention benefits of VMMC and generated a healthy competition among sites to see which site could help avert the most new HIV infections.

Finally, live capture and reporting of service delivery data through a web-based data collection system (and the use of short message service text messaging to campaign headquarters when Internet access was a challenge) helped inform the campaign leadership about trends in service delivery numbers, so that necessary adjustments to the deployment of personnel (e.g., increased deployment of counselors to the highest-volume sites) could be made in a timely manner.

Tanzania's application of select approaches illustrates how innovations can lead to successful VMMC scale-up programs. However, rapid scale-up may also have its downsides. Tanzania does not currently have enough data to confidently measure the effect of VMMC scale-up on its existing health services, but because the scale-up uses public health facilities, it is likely to increase the work burden on the public sector employees and stretch the provision of other services at health facilities.

### Kenya Case Study: Empowerment of the Nursing Workforce to Provide VMMC Services Increases Coverage while Maintaining Client Safety

In Kenya, the majority of men are already circumcised for cultural or religious reasons, but in Nyanza Province only 48% of men are circumcised. Nyanza is the epicenter of the Kenyan HIV epidemic, with an adult HIV prevalence of 14.9%—more than double the national average of 7.1%. Moreover, 13.2% of uncircumcised men in Kenya are HIV-positive, compared to just 3.9% of circumcised men [25].

Kenya is the first country in southern and eastern Africa to reach scale in its VMMC program. Between its formal launch in October 2008 and June 2011, the Kenyan VMMC program conducted 318,000 VMMCs, most of them in Nyanza Province [22]. Two key innovations contributed to this high volume of VMMCs. First, the national and provincial VMMC task forces set an ambitious target of 860,000 VMMCs nationally by 2013, including 426,500 in Nyanza Province. Targets were also established at the district level [26]. Second, task shifting to nurses was introduced to increase the number of VMMC providers. When the VMMC program was launched, the nursing scope of practice in Kenya did not cover VMMC; only medical doctors and clinical officers were legally authorized to provide VMMC surgery. In 2008, a facility readiness assessment conducted in Nyanza Province found that while doctors and clinical officers were in short supply at most health facilities, 85% of facilities had sufficient nurses to provide VMMC services [27]. In June 2009, the Ministry of Health announced a new policy enabling nurses to provide VMMC surgical services, and VMMC implementing partners such as the Nyanza Reproductive Health Society began to train nurses to provide the entire surgery. Data to date show no differences in adverse events rates in VMMCs performed by nurses compared to clinical officers or doctors, as long as providers have enough practice to reach competency [28].

Following the 2009 policy shift, the Kenya VMMC program was able to conduct two successful Rapid Results Initiatives. In November and December 2009, more than 36,000 VMMCs were conducted; in November and December 2010, another 50,000 men and adolescent boys received VMMC [29]. It is highly unlikely that this volume of services could have been achieved if the program had had to rely on doctors and clinical officers to provide VMMC surgery. However, although task shifting to nurses has improved the efficiency of VMMC sites, the removal of nurses from their normal public sector health facilities to participate in VMMC campaigns has almost certainly affected the delivery of other health services and programs.

### Swaziland Case Study: Preparing to Meet the Human Resource Requirements of an Accelerated Saturation Initiative

Circumcision is not a traditional practice in Swaziland, which has the highest adult HIV prevalence in the world (26%) [6]. In 2006–2007, only 8.2% of Swazi men reported being circumcised [30]. In 2009, Swaziland finalized its national VMMC strategy [31], which outlined a plan for providing VMMC to 144,688 males within five years. However, after hearing about the success of Kenya's 2009 Rapid Results Initiative, the Swaziland Male Circumcision Task Force decided to reconsider its timeline for VMMC scale-up. Termed the “Accelerated Saturation Initiative” in English and *Soka Uncobe* (Circumcise and Conquer) in SiSwati, the new initiative, which was formally launched on July 15, 2011, aims to complete VMMC scale-up in less than two years. Modeling studies suggest that while a five-year VMMC scale-up would avert an estimated 66,000 new HIV infections in Swaziland by 2025, accelerating the scale-up to one year would avert 88,000 new infections over the same time period [6].

Several approaches have been taken to meet the HRH requirements of *Soka Uncobe*. The first step was to establish an Human Resources Subcommittee within the national VMMC task force. This subcommittee worked with the Clinical Subcommittee to determine a staffing pattern for high-volume, high-efficiency VMMC teams capable of providing 40 VMMCs per day at multi-bed sites (Table 2). This staffing pattern was informed by the experiences of other VMMC programs, including the Iringa Region VMMC campaign in Tanzania, but includes an additional counselor in the form of an HIV-positive “expert client” who provides additional post-test counseling and linkages to care and treatment for prospective VMMC clients who test HIV-positive.

**Table 2:** Composition of VMMC clinical teams in Swaziland initiative.

Title	Number	Role on Team
Site manager	1	Ensure that the team has all supplies and equipment necessary, supervise team members and coordinate with demand-generation partners
Doctor	1	Remove foreskin, achieve hemostasis, place horizontal and vertical mattress sutures
Anesthesia/suture nurse	1	Apply local anesthesia, place simple interrupted sutures
Bedside nurses	4	Prep and drape client, assist during MC surgery, place bandage
Recovery room nurse	1	Monitor vital signs, provide postoperative care instructions, check bandage before discharging client
Review nurse	1	Conduct two-day and seven-day reviews
Counselors	3	Conduct group education, MC counseling, and HTC
Runner (non-clinical)	1	Ensure that each surgical bay has the supplies it needs
Hygienist	1	Ensure the clinic is clean, assist with waste management (only one needed due to use of fully disposable MC instrument kits and waste management company)
Expert client	1	Link clients who test HIV-positive to care and treatment services, model adherence to antiretroviral therapy
Receptionist	1	Book clients, start client record form
Data capturer	1	Record client data for monitoring and evaluation purposes
Booking agent	1	Manage client booking and link client with transport

The Human Resources Subcommittee determined that all non-clinical staff, including counselors, could be recruited and trained from within local communities. However, to limit the disruption to other key health services during *Soka Uncobe*, it decided that the majority of doctors would need to be recruited as volunteers from outside of Swaziland. Pilot testing of a formal “VMMC volunteer doctor program” began in April 2010, when four urologists from the American Urological Association visited Swaziland for a two-week period to provide VMMC services at three sites. During this pilot trip and two subsequent volunteer doctor visits, 17 doctors from Cameroon, Ethiopia, Ghana, Lesotho, and the United States performed 2,935 VMMCs. Before deployment, all visiting doctors have to complete an online learning tool and send documentation of their qualifications and licensure for review and registration by the Swaziland Medical and Dental Council. Upon arrival in Swaziland, their history taking, clinical examination, and surgical skills are validated through an observed structured clinical examination before they proceed to supervised clinical practice, at which time their skills in the forceps-guided technique are standardized by a full-time *Soka Uncobe* doctor.

Turning to nurses, who are allowed to inject local anesthesia, place simple interrupted sutures and conduct preoperative and postoperative exams in Swaziland (task sharing), the Human Resources Subcommittee initially assumed it would be impossible to identify enough nurses from within Swaziland to meet the needs of VMMC scale-up given the nursing shortages in the Swazi public sector. At the peak of *Soka Uncobe*, up to 245 nurses will be needed to staff up to 35 teams, 182 of whom will be in addition to nurses already working full-time in the national VMMC program. However, quantification of the existing nursing workforce in Swaziland by the Human Resources Subcommittee revealed that there are sufficient Swaziland-based nurses to staff *Soka Uncobe* and that these nurses are mainly “unemployed but registered” and “on leave” (Table 3). The “unemployed but registered” category consists primarily of foreign (mainly Zimbabwean) nurses. Because foreign nurses are not prioritized for civil service positions, many of them remain unemployed. The “on leave” category includes nurses on facility-approved and officially scheduled vacations.

**Table 3:** Quantification of the nursing workforce in the Kingdom of Swaziland.

Category	Number	Comments
Unemployed but registered with the Swaziland Nursing Council	110	Zimbabwean: 57 (51.8%); Swazi: 32 (29.1%); Zambian: 4 (3.64%); Congolese: 4 (3.64%); Ugandan: 1 (0.9%); Ghanaian: 1 (0.9%); Nigerian: 1 (0.9%); Unknown: 10 (9.1%)
Recently retired (within five years)	12	These nurses all indicated their interest in returning to work to support MC services
Newly graduating professional nurses	8	Eight graduating nurses indicated their interest in working on MC full-time
Swazi nurses working in the UK	5	These nurses all indicated their interest in returning to Swaziland work to support *Soka Uncobe*
Newly graduating nursing assistants	17	These nursing assistants can staff recovery rooms or serve as bedside nurses (not anesthesia/suture nurses)
On-leave from public sector employment	Average of 107 per month	Most of these nurses are on vacation, not medical or maternity leave
Total	259	

It should be noted that the recruitment out-of-workforce nurses for *Soka Uncobe* has some important limitations. Because nurses will need to take leave with preapproval from their employer to participate in VMMC scale-up, any change in the timing or location of the VMMC campaign might mean on-leave nurses will not be in the right place at the right time to work in the campaign. Thus, a dependency on on-leave nurses would render the VMMC scale-up program less able to change course to respond to demand. In addition, targeted recruitment of out-of-workforce nurses will provide sufficient nurses for *Soka Uncobe* only if all new graduated nurses enter government employment. This is an unrealistic expectation, as new graduates may pursue other options or not enter the workforce.

## Conclusions

Although the HRH shortages in the 14 VMMC priority countries/regions will probably not be resolved by 2015, when the suggested target of 80% VMMC coverage is due, the seven approaches that we discuss here (surgical efficiencies, non-surgical efficiencies, and five human resource efficiencies) highlight innovative solutions to HRH challenges. Moreover, the three case studies that we present suggest that it is possible to achieve good results in this early phase of VMMC scale-up in southern and eastern Africa in a short time frame by developing and implementing new approaches to reduce HRH shortages that aim to minimize disruptions to other health services. Importantly, the case studies demonstrate various options for addressing HRH challenges in different policy environments.

It is unlikely that the seven approaches we have identified are the only approaches being used to deal with the HRH challenges associated with VMMC scale-up in southern and eastern Africa, and further work, in the form of a systematic review, may identify more approaches. In addition, most of the evidence we reviewed came from programmatic and policy reports rather than the peer-reviewed literature and must therefore be considered with some caution. Of most concern, while it can be expected that campaigns that use existing public health facilities and public sector employees will add a burden to health service facilities and manpower, we found no published data on the repercussions of VMMC scale-up on other health programs. VMMC campaigns could have an effect on other health initiatives by diverting public focus, health workers, and government energy and spending. This is an area for future study that should be tackled before the approaches described in the three case studies are widely applied.

While we found evidence for success in dealing with HRH shortages during VMMC scale-up in three countries, there is still considerable work to be done in all 14 VMMC priority countries/regions. To perform the 20.33 million circumcisions between 2011 and 2015 that are required to reach 80% coverage, all the priority countries need to look at innovations, best practices, and strategies in the areas of surgical efficiencies, non-surgical efficiencies, and human resource efficiencies. The approaches and case studies discussed in this review can be adapted and refined by these other countries, but additional new approaches that address HRH challenges of VMMC scale-up specific to each country will need to be developed. Finally, we suggest that the seven approaches we have identified for meeting the human resource needs of VMMC scale-up, and others not revealed by our program review, will also be relevant for other high-impact health interventions that are currently facing HRH challenges.

## Abbreviations

HTC: HIV testing and counseling
HRH: human resources for health
MC: male circumcision
PEPFAR: United States President's Emergency Plan for AIDS Relief
UNAIDS: Joint United Nations Programme on HIV/AIDS
VMMC: voluntary medical male circumcision
WHO: World Health Organization
